# Type I Interferon Signaling Is Required for CpG-Oligodesoxynucleotide-Induced Control of *Leishmania major*, but Not for Spontaneous Cure of Subcutaneous Primary or Secondary *L. major* Infection

**DOI:** 10.3389/fimmu.2018.00079

**Published:** 2018-02-05

**Authors:** Ulrike Schleicher, Jan Liese, Nicole Justies, Thomas Mischke, Simone Haeberlein, Heidi Sebald, Ulrich Kalinke, Siegfried Weiss, Christian Bogdan

**Affiliations:** ^1^Mikrobiologisches Institut – Klinische Mikrobiologie, Immunologie und Hygiene, Friedrich-Alexander-Universität (FAU) Erlangen-Nürnberg and Universitätsklinikum Erlangen, Erlangen, Germany; ^2^Medical Immunology Campus Erlangen, FAU Erlangen-Nürnberg, Erlangen, Germany; ^3^Abteilung Mikrobiologie und Hygiene, Institut für Medizinische Mikrobiologie und Hygiene, Universitätsklinikum Freiburg, Freiburg, Germany; ^4^Institut für Experimentelle Infektionsforschung, TWINCORE, Zentrum für Experimentelle und Klinische Infektionsforschung, eine Gemeinschaftseinrichtung vom Helmholtz Zentrum für Infektionsforschung und der Medizinischen Hochschule Hannover, Hannover, Germany; ^5^Abteilung für Molekulare Immunologie, Helmholtz Zentrum für Infektionsforschung, Braunschweig, Germany

**Keywords:** *Leishmania major*, type I interferon, interferon-alpha/beta, innate immunity, cutaneous leishmaniasis

## Abstract

We previously showed that in mice infected with *Leishmania major* type I interferons (IFNs) initiate the innate immune response to the parasite at day 1 and 2 of infection. Here, we investigated which type I IFN subtypes are expressed during the first 8 weeks of *L. major* infection and whether type I IFNs are essential for a protective immune response and clinical cure of the disease. In self-healing C57BL/6 mice infected with a high dose of *L. major*, IFN-α4, IFN-α5, IFN-α11, IFN-α13, and IFN-β mRNA were most prominently regulated during the course of infection. In C57BL/6 mice deficient for IFN-β or the IFN-α/β-receptor chain 1 (IFNAR1), development of skin lesions and parasite loads in skin, draining lymph node, and spleen was indistinguishable from wild-type (WT) mice. In line with the clinical findings, C57BL/6 IFN-β^−/−^, IFNAR1^−/−^, and WT mice exhibited similar mRNA expression levels of IFN-γ, interleukin (IL)-4, IL-12, IL-13, inducible nitric oxide synthase, and arginase 1 during the acute and late phase of the infection. Also, myeloid dendritic cells from WT and IFNAR1^−/−^ mice produced comparable amounts of IL-12p40/p70 protein upon exposure to *L. major in vitro*. In non-healing BALB/c WT mice, the mRNAs of IFN-α subtypes (α2, α4, α5, α6, and α9) were rapidly induced after high-dose *L. major* infection. However, genetic deletion of IFNAR1 or IFN-β did not alter the progressive course of infection seen in WT BALB/c mice. Finally, we tested whether type I IFNs and/or IL-12 are required for the prophylactic effect of CpG-oligodesoxynucleotides (ODN) in BALB/c mice. Local and systemic administration of CpG-ODN 1668 protected WT and IFN-β^−/−^ mice equally well from progressive leishmaniasis. By contrast, the protective effect of CpG-ODN 1668 was lost in BALB/c IFNAR1^−/−^ (despite a sustained suppression of IL-4) and in BALB/c IL-12p35^−/−^ mice. From these data, we conclude that IFN-β and IFNAR1 signaling are dispensable for a curative immune response to *L. major* in C57BL/6 mice and irrelevant for disease development in BALB/c mice, whereas IL-12 and IFN-α subtypes are essential for the disease prevention by CpG-ODNs in this mouse strain.

## Introduction

The term “type I interferons” is used for a multigene family of cytokines that in the mouse comprises 14 interferon (IFN)-α genes encoding proteins and single genes of IFN-β, IFN-κ, and IFN-ε (1). All type I IFNs signal *via* a common IFN-α/β-receptor (IFNAR) complex, which consists of two chains (IFNAR1 and IFNAR2) (2). Type I IFNs were originally described for their antiviral activity, which is due to the induction of genes that degrade mRNA, impair protein synthesis, help to sequester viral nucleocapsids, induce cytoplasmic viral nucleic acid detecting receptors, or amplify the IFN response (3–5). Since then it has become clear that type I IFNs also have numerous immunomodulatory functions, which include both activating and inhibitory effects on macrophages, dendritic cells (DCs), natural killer (NK) cells, T lymphocytes, and B cells (5–8). Therefore, it was not surprising to see that in mice exposed to certain non-viral pathogens (bacteria, protozoa, fungi, or helminths) or microbial products a deficiency of IFN-α/β signaling or the application of type I IFNs positively or negatively influenced the outcome of the infection [reviewed in Ref. (9–11)]. To date, there is little information available on the expression and differential activities of the type I IFN subtypes during non-viral infections *in vivo*. In general, IFN-β is assumed to play a master role, as in many cell types it induces and amplifies the expression of IFN-α genes and thereby dominates the entire type I IFN response (12, 13); however, IFN-β-independent production of IFN-α has also been described (14, 15).

*Leishmania* promastigotes are flagellated protozoan parasites that under natural conditions are transmitted to mammalian organisms by the bite of sand flies. Infections can lead to cutaneous, mucocutaneous or visceral disease depending on the parasite species and strain, the infection inoculum, and the immune response of the host. The experimental infection of different inbred strains of mice with *Leishmania* (subgenus *Leishmania*) *major* (in the following abbreviated as *L. major*) has proven to be a useful model for self-healing vs. non-healing cutaneous leishmaniasis and for the analysis of the components of the immune system that are required for parasite control and resolution of the infection. Previous studies showed that interleukin (IL)-12, IFN-γ, tumor necrosis factor (TNF), inducible or type 2 nitric oxide synthase [iNOS (NOS2)], and CD4^+^ type 1 T helper (Th1) cells are essential for overcoming an infection with *L. major* (16–18).

With respect to type I IFNs, earlier results pointed to a protective function in mouse *L. major* infections. First, *in vitro* simultaneous exposure of mouse macrophages to purified IFN-α/β and *L. major* promastigotes led to expression of iNOS and subsequent killing of intracellular amastigotes (19). Similarly, human mononuclear phagocytes acquired antimicrobial activity against *L. major* amastigotes after stimulation with IFN-β (20). Second, short-term neutralization of IFN-α/β immediately before cutaneous infection with *L. major* strongly reduced the expression of iNOS protein, the activation of NK cells, the expression of IFN-γ mRNA, and the containment of the parasites at days 1 and 2 of infection in the skin and draining lymph node (dLN) (21). Third, prophylactic treatment of otherwise non-healing BALB/c mice with low doses of recombinant mouse IFN-β protected the majority of the mice from progressive disease (22). However, so far it has not been analyzed which type I IFN subtypes are expressed during the course of *L. major* infection. Furthermore, it is unknown whether endogenous type I IFNs are required for the control of primary or secondary *L. major* infections in self-healing C57BL/6 mice or for the previously reported (23) immunoprophylactic effect of CpG-oligodesoxynucleotides (ODN) in BALB/c mice. Finally, it has never been investigated whether type I IFN signaling contributes to the susceptibility of *L. major*-infected BALB/c mice as suggested by the disease-aggravating role of type I IFNs observed in infections with other *Leishmania* species (24–26).

In this study, we were able to address these issues with the help of recombinant mice that were deficient for IFN-β (IFN-β^−/−^ mice) or type I IFN signaling (IFNAR1^−/−^ mice) and that were thoroughly backcrossed to the C57BL/6 or BALB/c background.

## Materials and Methods

### Mice

Wild-type (WT) C57BL/6 and BALB/c mice were purchased from Charles River Breeding Laboratories (Sulzfeld, Germany). IFNAR1^−/−^ mice, which were originally generated on a 129/SvEv background (27), were backcrossed to C57BL/6 for more than 20 generations (B6.IFNAR1^−/−^) by one of us (UK) at the Paul-Ehrlich-Institute, Langen, Germany. Breeding pairs of IFNAR1^−/−^ mice (27) backcrossed to BALB/c for seven generations (28) were kindly provided by Daniel Portnoy (University of California, Berkeley, USA) and were then used for further backcrossings to BALB/c background (BALB/c.IFNAR1^−/−^) for four generations using speed congenic technology (29). IFN-β^−/−^ mice (12) were backcrossed to C57BL/6 (B6.IFN-β^−/−^) or BALB/c background (BALB/c.IFN-β^−/−^) for 12 or 15 generations, respectively. IL-12p35^−/−^ mice backcrossed to C57BL/6 background for 11 generations were obtained from the Jackson Laboratories (Bar Harbor, ME, USA). Breeding pairs of IL-12p35^−/−^ mice (30) backcrossed to BALB/c background for five generations were generously supplied by G. Alber (University of Leipzig, Germany).

Wild-type and knockout mice were age- and sex-matched in the experiments. All mice were kept under specific pathogen-free conditions in the facilities of (a) the Institute of Medical Microbiology and Hygiene at the University Hospital Freiburg, (b) the Microbiology Institute at the University Hospital Erlangen, or (c) the Franz-Penzoldt-Zentrum for Animal Research, Friedrich-Alexander-University of Erlangen-Nürnberg. The infection experiments were approved by the governmental animal-welfare committees of the Regierungspräsidium Freiburg, Germany and of the Government of Middle Franconia, Ansbach, Germany.

### Parasites and Infection

The origin of the *L. major* strain MHOM/IL/81/FEBNI was described before (31, 32). Promastigotes were maintained *in vitro* in RPMI1640 plus 10% FCS on Novy–Nicolle–MacNeal (NNN) rabbit blood agar slants for a maximum of six passages. Fresh *L. major* promastigotes were derived from amastigotes that were isolated from non-ulcerated skin lesions of infected BALB/c mice (33). For *in vitro* expansion, *L. major* promastigotes were transferred from the NNN-cultures into complete Schneider’s *Drosophila* insect cell medium [Genaxxon Bioscience; supplemented with 10% (v/v) heat-inactivated FCS, 10 mM HEPES, 1 mM sodium pyruvate, 2 mM l-glutamine, 0.27 mM l-asparagine, 0.55 mM l-arginine, 100 U/ml penicillin G, 100 µg/ml streptomycin, and 2% (v/v) normal human urine in modification of previous protocols (34, 35)] and grown to stationary phase. Mice were infected with 3 × 10^6^ stationary phase *L. major* promastigotes (derived from NNN-cultures) in 50 µl of PBS subcutaneously (s.c.) into one or both hind footpads. For control purposes, mice were injected with PBS alone in some experiments. Footpad swelling was determined once or twice weekly with a metric caliper (in mm; Kroeplin, Schlüchtern, Germany). The relative footpad thickness increase was calculated in relation to the other footpad (in unilateral infection experiments) or the footpad thickness before infection (in bilateral primary infection experiments or after secondary infection).

### Quantification of Parasite Burdens

Tissue parasite burdens were determined by limiting dilution analysis using twofold, threefold, or fivefold dilution steps and 12 replicates per dilution in complete Schneider’s *Drosophila* medium (see above) as described before (33, 36). The statistical analysis was performed with the L-Calc™ (StemSoft Software, Vancouver, BC, Canada) or ELIDA software, which analyses data by applying the Poisson distribution and by the χ^2^ test (37). Statistical significance was assumed when 95% confidence intervals did not overlap.

### Treatment of Mice with CpG-ODNs

Following a published protocol (23), BALB/c mice (WT, IFN-β^−/−^, or IFNAR1^−/−^) were treated twice with 10 nmol CpG-ODN 1668 or 2216 (Thermo Electron, Ulm, Germany), i.e., 2 h before and 10 h after the infection with *L. major* promastigotes. At each time-point, half of the dose (5 nmol) was given s.c. at the site of infection in 40 µl PBS (20 µl per footpad in experiments with bilateral infection), while the other half was administered intraperitoneally (i.p.) in a volume of 500 µl PBS. Alternatively, the total applied dose of CpG-ODN was reduced to 10 nmol (with 5 nmol given s.c. at 2 h before infection and 5 nmol injected i.p. at 10 h after infection), which was equally effective in protecting WT BALB/c mice. Control mice received PBS alone.

### Gene Expression Analysis

Excised organs and tissues were directly stored in RNAlater reagent (Qiagen, Hilden, Germany) for at least 24 h. Organs were then homogenized in a Mixer Mill MM 200 (Retsch, Haan, Germany) before extracting total RNA using TRIZOL reagent (Life Technologies Invitrogen, Darmstadt, Germany). Contaminating genomic DNA was removed with DNase (DNAfree, Life Technologies Ambion^®^). Presence of genomic DNA was excluded by performing a PCR reaction with 1 µl of the RNA sample as template and primers for mouse β-actin (*sense*: 5′-CACCCGCCACCAGTTCGCCA-3′; *antisense*: 5′-CAGGTCCCGGCCAGCCAGGT-3′). Five to ten micrograms of RNA were reverse transcribed with the High Capacity cDNA Reverse Transcription Kit (Life Technologies Applied Biosystems). For quantitative PCR, 100 ng of each cDNA was analyzed in triplicates using the ABI PRISM HT7900 system (Life Technologies Applied Biosystems) and the following gene-specific assays (TaqMan Gene Expression Assays; Life Technologies Applied Biosystems): mIFN-α2 (Mm00833961_s1), mIFN-α4 (Mm00833969_s1), mIFN-α5 (Mm00833976_s1), mIFN-α6 (Mm01258374_s1), mIFN-α9 (Mm00833983_s1), mIFN-α11 (Mm01257312_s1), mIFN-α12 (Mm00616656_s1), mIFN-α13 (Mm00781548_s1), mIFN-α14 (Mm01703465_s1), mIFN-β (Mm00439546_s1), mIFN-γ (Mm00801778_m1), mIL-2 (Mm00434256_m1), mIL-4 (Mm00445259_m1), mIL-10 (Mm00439616_m1), mIL-12p35 (Mm00434165_m1), mIL-12p40 (Mm00434170_m1), mIL-13 (Mm00434204_m1), mIL-15 (Mm00434210_m1), mIL-18 (Mm00434225_m1), IL-23p19 (Mm00518984_m1), murine iNOS (NOS2) (Mm00440485_m1), and murine arginase 1 (Arg1) (Mm00475988_m1). The gene for mouse hypoxanthine guanine phosphoribosyl transferase 1 (mHPRT-1; Mm00446968_m1) was used as an endogenous control for quantification of mRNA levels. All mRNA levels were determined in duplicates or triplicates with the help of the SDS 2.3 Software (Life Technologies Applied Biosystems^®^). Relative expression levels were calculated using the following formula: relative expression = 2^−(Ct(Target Gene)−Ct(Endogenous Control))^ × *f*, with *f* = 10^4^ as an arbitrary factor.

### DCs and Stimulation by *Leishmania* and CpG-ODNs

Bone marrow (BM) was isolated from hind legs of naïve WT, IL-12p35^−/−^ or IFNAR1^−/−^ mice after anesthesia and subsequent cervical dislocation. Femoral and tibial bones were opened on both sides under sterile conditions, and bone marrow cells were flushed out with PBS using a 27G hollow needle.

Bone marrow-derived conventional (or myeloid) DCs (cDCs or mDCs) were differentiated by culturing 6 × 10^6^ BM cells in RPMI1640 medium [containing 2 mM l-glutamine, 10 mM HEPES, 50 µM 2-mercaptoethanol, 100 U/ml penicillin G, 100 µg/ml streptomycin, 10% (v/v) FCS, and 10% (v/v) culture supernatant (SN) of Ag8653 myeloma cells transfected with a murine GM-CSF expression plasmid (38)]. BM cells were cultured for 7 days in 60 cm^2^ culture dishes with initially 10 ml of medium, before on days 3 and 6, 10 ml fresh medium was added. mDC cultures contained 60–80% CD11b^+^CD11c^+^ mDC on day 7 and were purified as immature CD11b^+^CD11c^+^CD86^−^ cells by flow cytometric cell sorting (MoFlo) (purity of >99%).

Bone marrow-derived plasmacytoid dendritic cells (pDCs) were generated from total BM cells in the presence of Flt3 ligand (39). After incubation in red blood cell lysis buffer for 5 min, cells were washed twice with 20 ml PBS. Bone marrow cells were cultured in 5 ml RPMI1640 [containing 2 mM l-glutamine, 1× non-essential amino acids, 1 mM sodium pyruvate, 100 µg/ml kanamycin, 50 µM 2-mercaptoethanol, 10% (v/v) FCS, and 50 ng/ml rmFlt3L (R&D Systems, Wiesbaden, Germany)] for 7–8 days at 2 × 10^6^ cells/ml in 25 cm^2^ cell culture flasks. At day 4, 2.5 ml of the culture medium was exchanged against fresh medium with 25 ng/ml Flt3L. After 7–8 days, 10–20% of the cells were B220^+^CD11b^int^CD11c^+^, and pDCs were further purified by MoFlo sorting gating on B220^+^CD11b^int^CD11c^+^ cells (purity >99%).

For ELISA and IFN-α/β bioassay studies, MoFlo™-sorted pDC and mDC were cultured in 96-well culture plates (10^5^ cells/well in 250 µl) using the respective pDC or mDC culture medium without growth factors. Cells were stimulated for 48 h with CpG-ODN 2216 (1 µM), CpG-ODN 1668 (1 µM), LPS (200 ng/ml), or *L*. *major* promastigotes (stationary growth phase; multiplicity of infection 3). SNs were harvested and stored at −20°C.

### Cytokine Measurements

Interferon-α/β levels were determined with an L929/vesicular stomatitis virus-protection assay using triplicates and serial twofold dilutions of the culture SNs (21). Purified mouse IFN-α/β and a neutralizing sheep-anti-IFN-α/β antiserum (provided by I. Gresser, Institute Curie, Paris) were used as a standard or to ascertain that all antiviral activity in the SNs was due to IFN-α/β. The content of TNF (eBiosciences, sensitivity 40 pg/mL), IL-12p40, or IL-12p70 (BD Biosciences, sensitivity 40 pg/mL) was measured by ELISA.

### Statistics

Statistical significance was analyzed using the non-parametric Mann–Whitney test. A *p* value <0.05 was considered significant.

## Results

### *L. major* Infection Leads to Differential Expression of Type I IFNs

**Figure 1:**
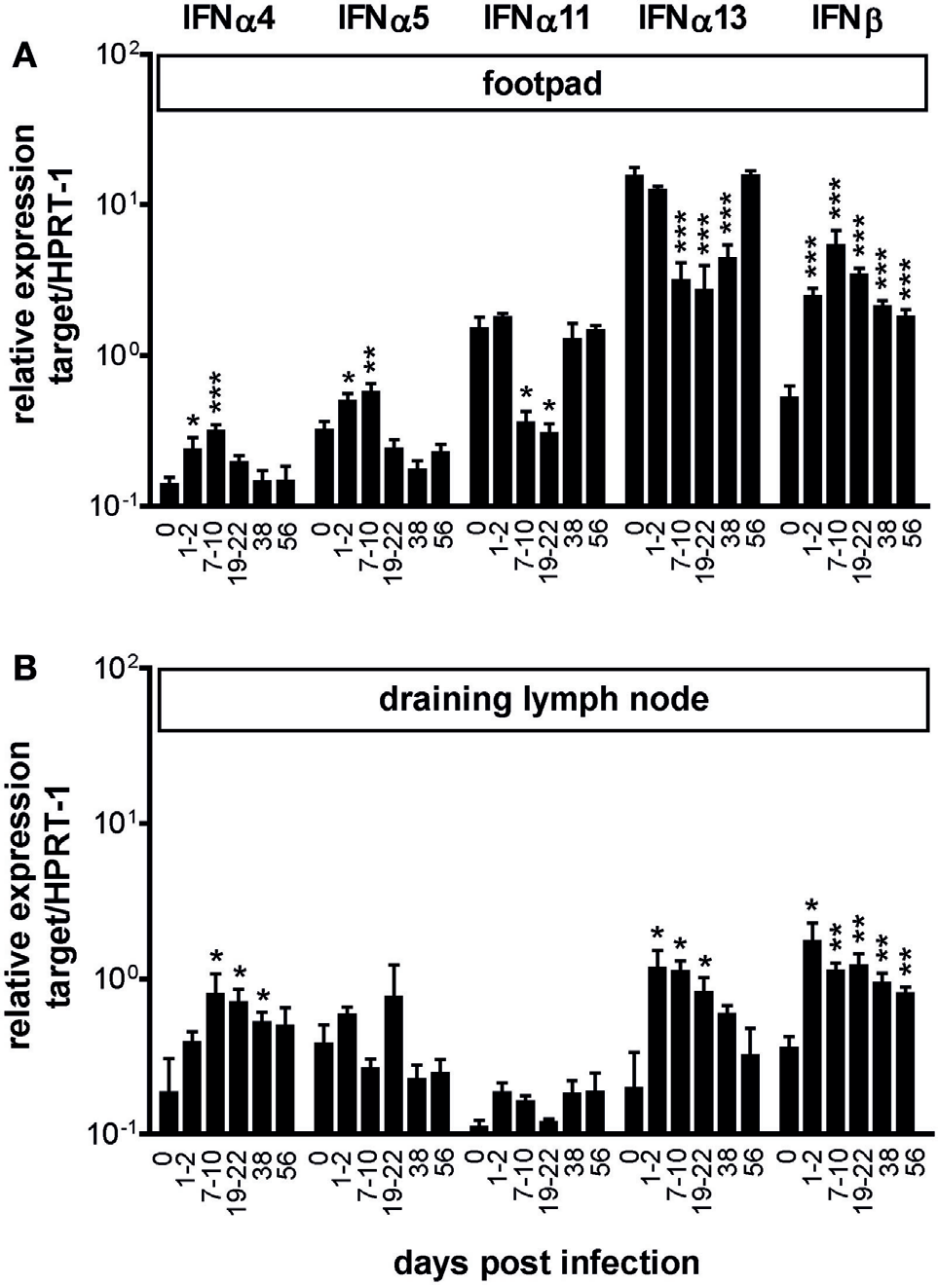
Expression of type I interferon (IFN) subtypes in *Leishmania major*-infected C57BL/6 mice. C57BL/6 wild-type mice were infected with 3 × 10^6^ stationary phase *L. major* promastigotes into the hind footpads or treated with PBS. The mRNA expression of type I IFN subtypes was analyzed in the footpad tissues **(A)** and in the draining lymph nodes **(B)** at different time-points after infection using quantitative RT-PCR (day 0 are naïve mice). Results are shown as mRNA levels (mean ± SEM) normalized to the endogenous control HPRT-1 from four independent experiments (three mice per group and time point, respectively). Asterisks represent the respective significance values compared with uninfected mice (*p < 0.05; **p < 0.01; and ***p < 0.001; Mann–Whitney test).

In a previous study, we reported the expression of IFN-α/β protein in skin lesions of C57BL/6 × 129/SvEv mice at 24 h after infection with *L. major*. The immunohistological analysis was restricted to this early time point and based on the use of a polyvalent anti-IFN-α/β antibody, which did not allow for differential detection of type I IFN subtypes (21). To obtain a more detailed view on type I IFN expression during the course of experimental cutaneous leishmaniasis (days 1–56), we performed quantitative mRNA expression analyses for several type I IFN family members in the skin lesions of C57BL/6 mice subcutaneously infected with *L. major*. Within 24–48 h of *L. major* infection the relative IFN-β mRNA expression level increased by a factor of 4.7 from 0.52 (±0.10) in uninfected mice to 2.45 (±0.34) in infected ones (mean ± SEM of seven independent experiments with two to six samples; *p* < 0.001 Mann–Whitney test). From week 1 of infection onward, the IFN-β mRNA remained on a high expression level until the end of the observation period (day 56), when the footpad lesions had already started to resolve (Figure 1A). Considerably weaker and more transient was the upregulation of IFN-α4 and IFN-α5 mRNA, which returned to baseline levels within 3–4 weeks of infection. IFN-α11 and especially IFN-α13 mRNA were constitutively expressed in the skin of naïve mice. Following infection, both IFN-α subtypes initially decreased but returned to normal levels after 6–7 weeks of infection (Figure 1A).

In the dLN, we also observed a temporary upregulation of the IFN-α4 and IFN-β mRNA expression in response to *L. major*. Unlike to our findings in the skin, however, the baseline IFN-α13 mRNA levels in the popliteal lymph nodes of naïve C57BL/6 mice were much lower and clearly increased during the first 4 weeks of infection (Figure 1B). In the spleen of *L. major*-infected C57BL/6 mice, the mRNA expression levels of the above-mentioned type I IFN subtypes remained largely constant throughout the infection period (data not shown). With respect to IFN-α6, IFN-α12, and IFN-α14, hardly any changes were observed in the three analyzed tissues, or mRNA expression was not detectable at all (data not shown).

To exclude that the injection procedure itself accounts for the early induction of type I IFNs in the skin and dLN, we compared PBS-injected (day 2) and *L. major*-infected C57BL/6 mice (day 2) with naïve C57BL/6 mice (day 0) for their expression of IFN-α5 and IFN-β mRNA. Injection of PBS alone already caused a slight, but significant induction of type I IFNs at both tissue sites. However, in the presence of *L. major*, the expression levels were significantly higher than in PBS-treated mice (Figure S1 in Supplementary Material).

Taking these data together, we conclude that an infection with *L. major* triggers a specific and primarily local type I IFN response with differential regulation of IFN-β and IFN-α subtypes.

### Type I IFNs Are Dispensable for the Control of *L. major* in Self-healing C57BL/6 Mice

**Figure 2:**
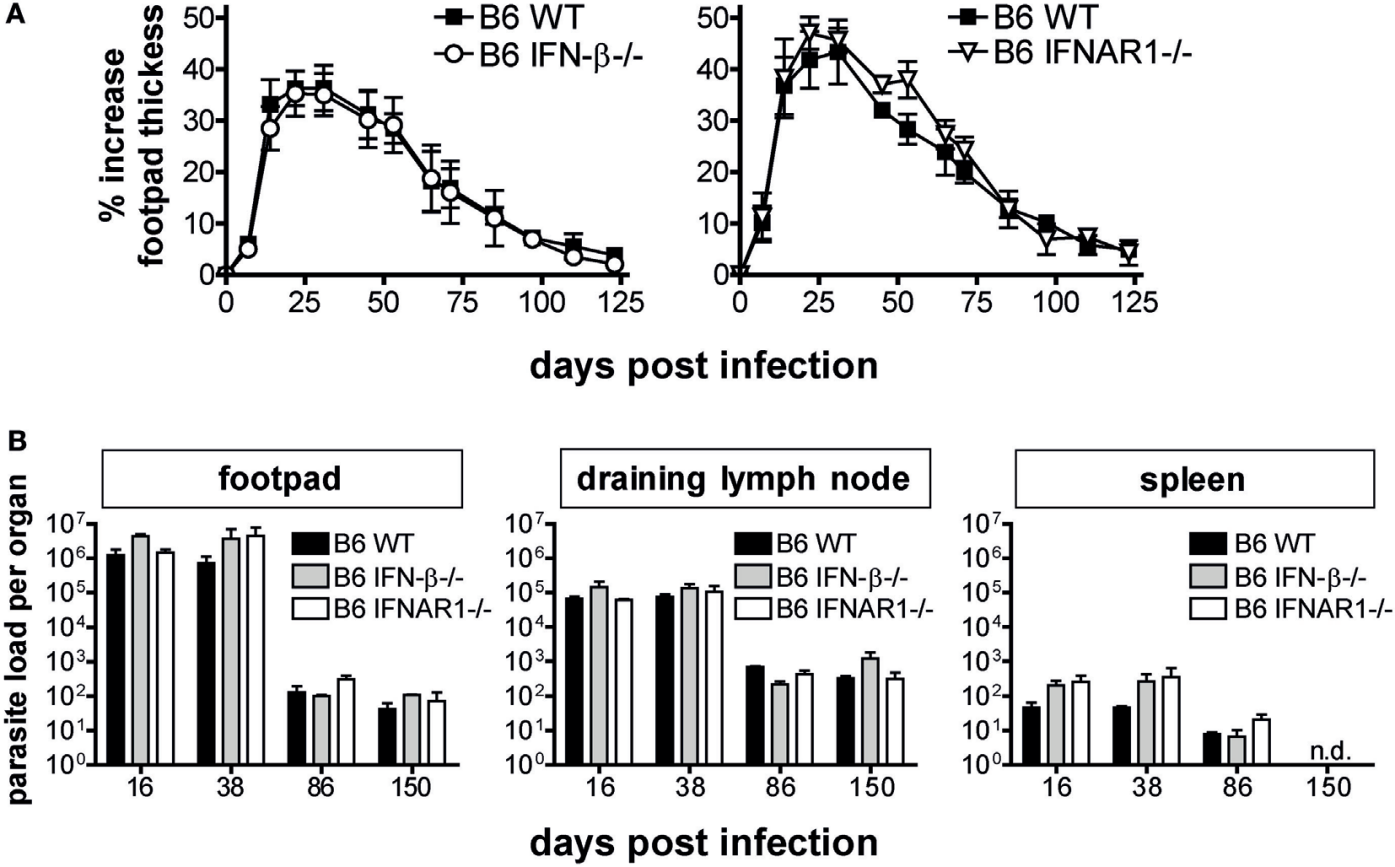
Interferon (IFN)-β and IFNAR signaling are dispensable for the control of primary infection with *Leishmania major* in C57BL/6 mice. **(A)** Development of footpad lesions in C57BL/6 wild-type (WT) vs. IFN-β^−/−^ vs. IFNAR1^−/−^ mice after infection with 3 × 10^6^ stationary phase *L. major* promastigotes into both hind footpads. The mean (±SEM) of the relative footpad thickness increase of 4 independent experiments with 9–12 mice per group is shown. **(B)** Parasite burden in the footpads, draining lymph nodes, and the spleens of C57BL/6 WT vs. IFN-β^−/−^ vs. IFNAR1^−/−^ mice. At the indicated time-points, three mice per group were analyzed for their parasite load in different organs by limiting dilution assays. The mean results (±SEM) of one representative out of four independent experiments **(A)** are presented. n.d., Not detectable.

**Figure 3:**
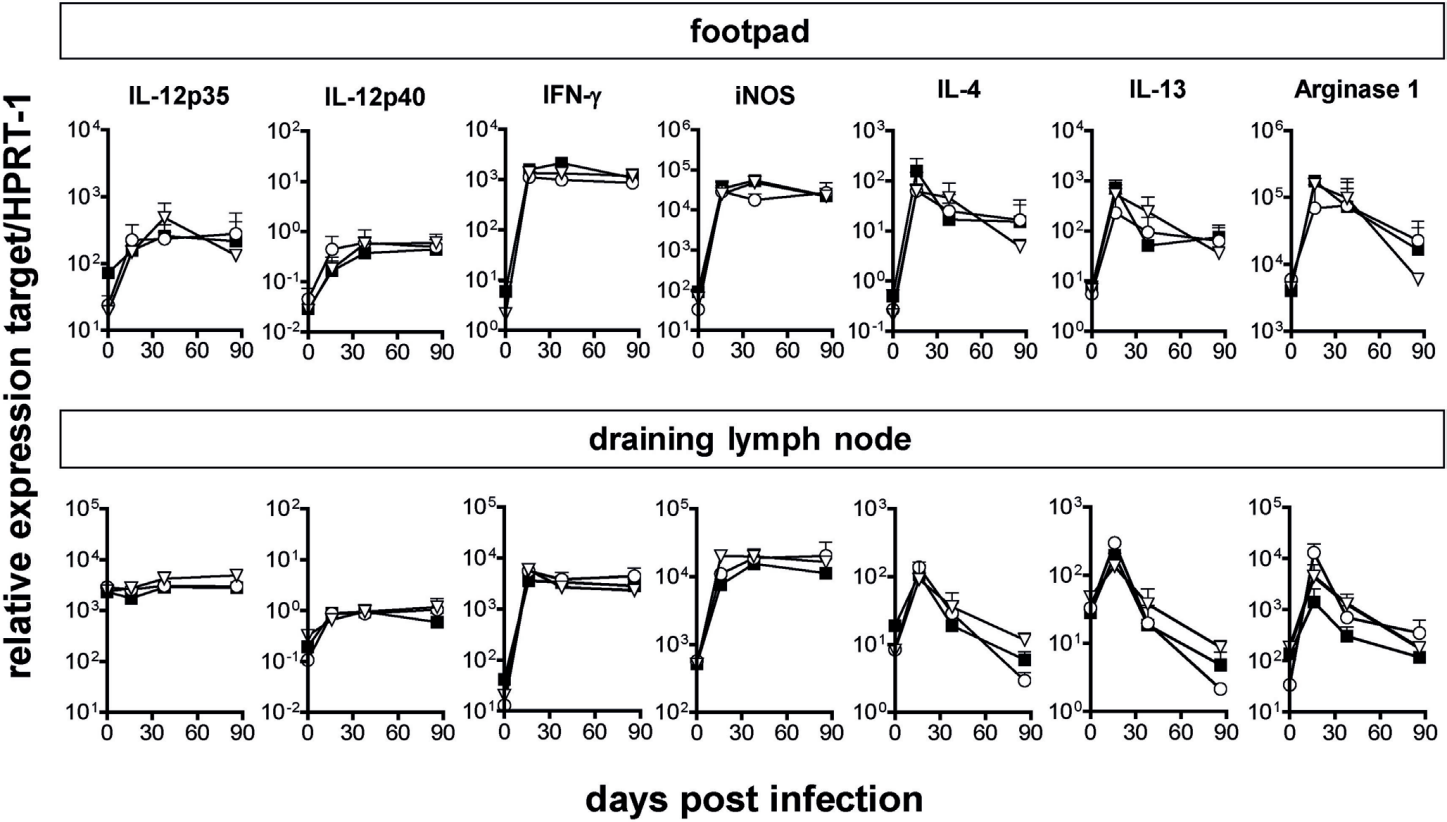
Comparable mRNA expression of cytokines and effector pathways in C57BL/6 wild-type (WT), IFN-β^−/−^, and IFNAR1^−/−^ mice during *Leishmania major* infection. Total RNA was isolated from footpad tissue or draining popliteal lymph nodes of WT (solid squares), IFN-β^−/−^ (open circles), and IFNAR1^−/−^ mice (open triangles) and reverse transcribed. Gene expression levels were determined by quantitative RT-PCR analysis using assays for the respective genes. Expression levels were calculated relative to the expression level of the endogenous control gene (HPRT-1). Results are mean expression levels from three mice per group and time point with error bars indicating SDs. The results of one of two experiments are shown.

The infection-dependent regulation of type I IFN expression led us to investigate a possible function of IFN for the outcome of *L. major* infections. In the light of the strong induction of IFN-β mRNA (Figure 1) and its known amplifying effect on the expression of other type I IFNs (40), we first investigated the course of *L. major* infection in C57BL/6 IFN-β^−/−^ mice. IFN-β^−/−^ mice controlled the clinical infection as efficiently as the respective WT mice (Figure 2A). Accordingly, the tissue parasite burdens in the skin lesions, dLNs, and spleens from WT and IFN-β^−/−^ mice were comparable (Figure 2B). Also, the time course of the mRNA expression of cytokines (IFN-γ, IL-12p35, IL-12p40, IL-4, and IL-13) and effector pathways (iNOS, arginase 1) that determine the quality of the anti-*Leishmania* immune response were virtually superimposable (Figure 3, solid squares vs. open circles).

The previous notion that IFN-β governs the entire type I IFN response has recently been challenged (14, 15, 41). To test the impact of the entire type I IFN family on an infection with *L. major*, we resorted to C57BL/6 mice that lack a functional type I IFN receptor. When C57BL/6 IFNAR1^−/−^ were infected with *L. major*, the clinical course of infection (Figure 2A), the parasite burden (Figure 2B), the cytokine mRNA expression pattern (IFN-γ, IL-12p35, IL-12p40, IL-4, and IL-13) at the site of infection and in the dLN, and the ability to mount a strong and persistent iNOS response along with a transient upregulation of arginase 1 (Figure 3, solid squares vs. open triangles) were indistinguishable in C57BL/6 WT and IFNAR1^−/−^ mice. After resolution of the skin swelling, we did not observe any clinical relapses in the mutant mice during an observation period of up to 241 days. Notably, when we infected IFNAR1^−/−^ mice [generated on a 129Sv/Ev background (27)] that had been backcrossed to C57BL/6 for only 6 instead of 20 generations, the size of the cutaneous lesions in the IFNAR1^−/−^ mice was significantly smaller than in the respective WT controls (data not shown). This observation presumably reflects incomplete backcrossing. Unlike to C57BL/6 mice that lack a mature and functional natural resistance-associated macrophage protein (NRAMP1^S^), 129Sv/Ev mice carry a fully functional NRAMP1 protein (NRAMP1^R^) and usually develop only minor skin lesions following *L. major* infection (42).

**Figure 4:**
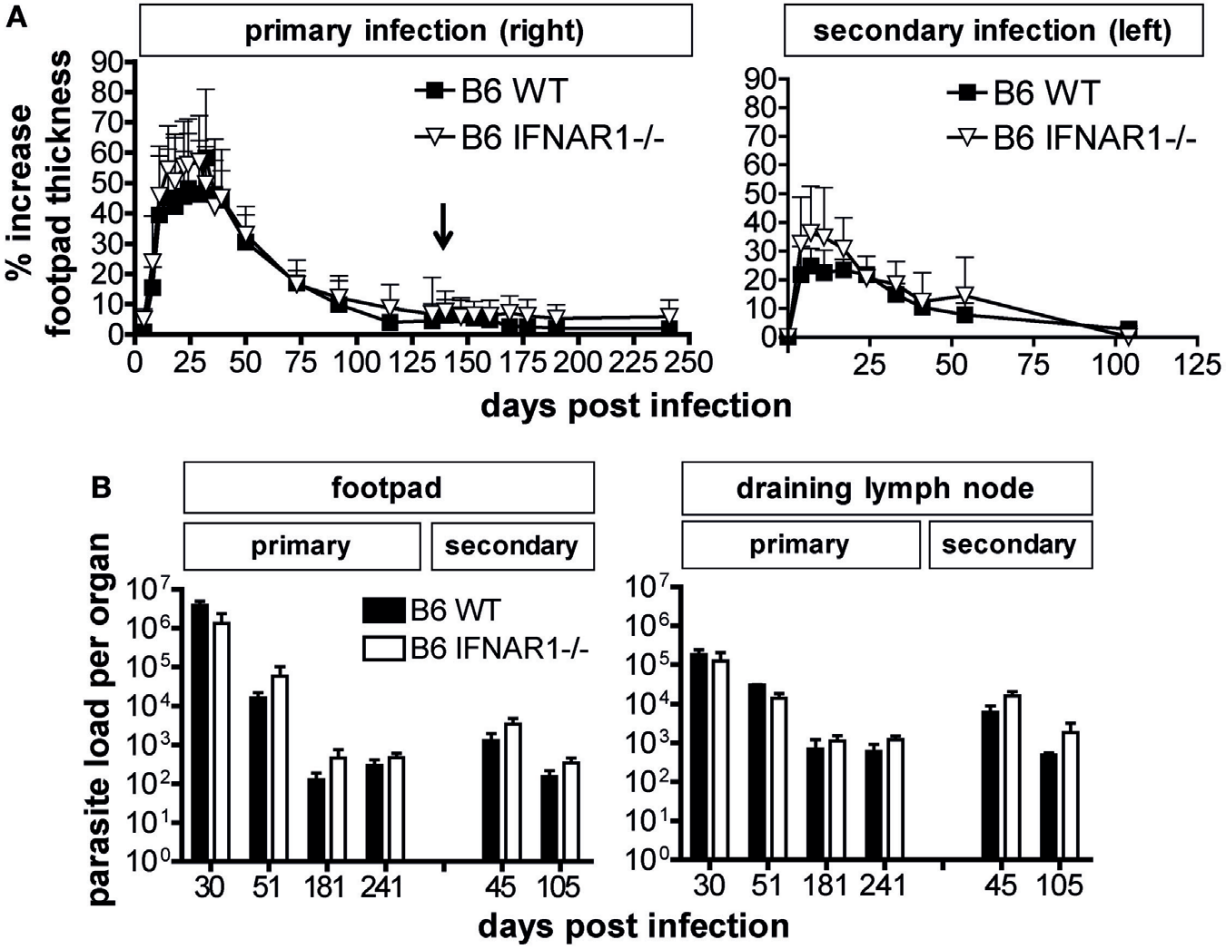
Comparable control of secondary *Leishmania major* infection in C57BL/6 wild-type (WT) and IFNAR1^−/−^ mice. For primary infection, mice were injected subcutaneously with 3 × 10^6^ stationary phase *L. major* promastigotes into the right hind footpad. At day 136 (indicated by ↓), i.e., after healing of the primary skin lesion, mice were reinfected with an identical parasite inoculum into the left hind footpad. **(A)** Clinical course of infection in C57BL/6 WT vs. IFNAR1^−/−^ mice. The mean (±SD) of the relative footpad thickness increase during primary (right footpad) and secondary infection (left footpad) is shown. One of three independent experiments with 12–18 mice per group is presented. In panel **(B)**, the tissue parasite burden in the right and left footpad and draining lymph node at various time points after primary (left) and secondary infection (right) is depicted (please note the different time scale of the abscissas). At the indicated time points, three mice per group were analyzed by limiting dilution assays. The mean results (±SEM) of one representative out of three independent experiments are shown.

As type I IFNs have been described to rescue activated or memory T cells from apoptosis and to increase the longevity of these cells (43–45), we considered the possibility that IFN-β^−/−^ and IFNAR1^−/−^ might only show a phenotype during secondary infection with *L. major*. We therefore challenged C57BL/6 WT and IFNAR1^−/−^ mice, which had healed a primary subcutaneous infection with *L. major*, by injection of the same parasite inoculum into the contralateral footpad. As expected based on earlier reports (46, 47), the secondary skin lesions of C57BL/6 WT mice were less severe and healed more rapidly than during the primary infection. Although there was a tendency for a disease aggravation in IFNAR1^−/−^ mice, the differences in lesion development (Figure 4A) and tissue parasite burden (Figure 4B) between C57BL/6 WT and IFNAR1^−/−^ mice were not significant in three independent experiments.

From these data, we conclude that both the control of a primary and of a secondary infection with *L. major* can occur independently of type I IFN signaling.

### Type I IFNs Are Dispensable for *L. major*-Induced Production of IL-12 by C57BL/6 Myeloid DCs

**Figure 5:**
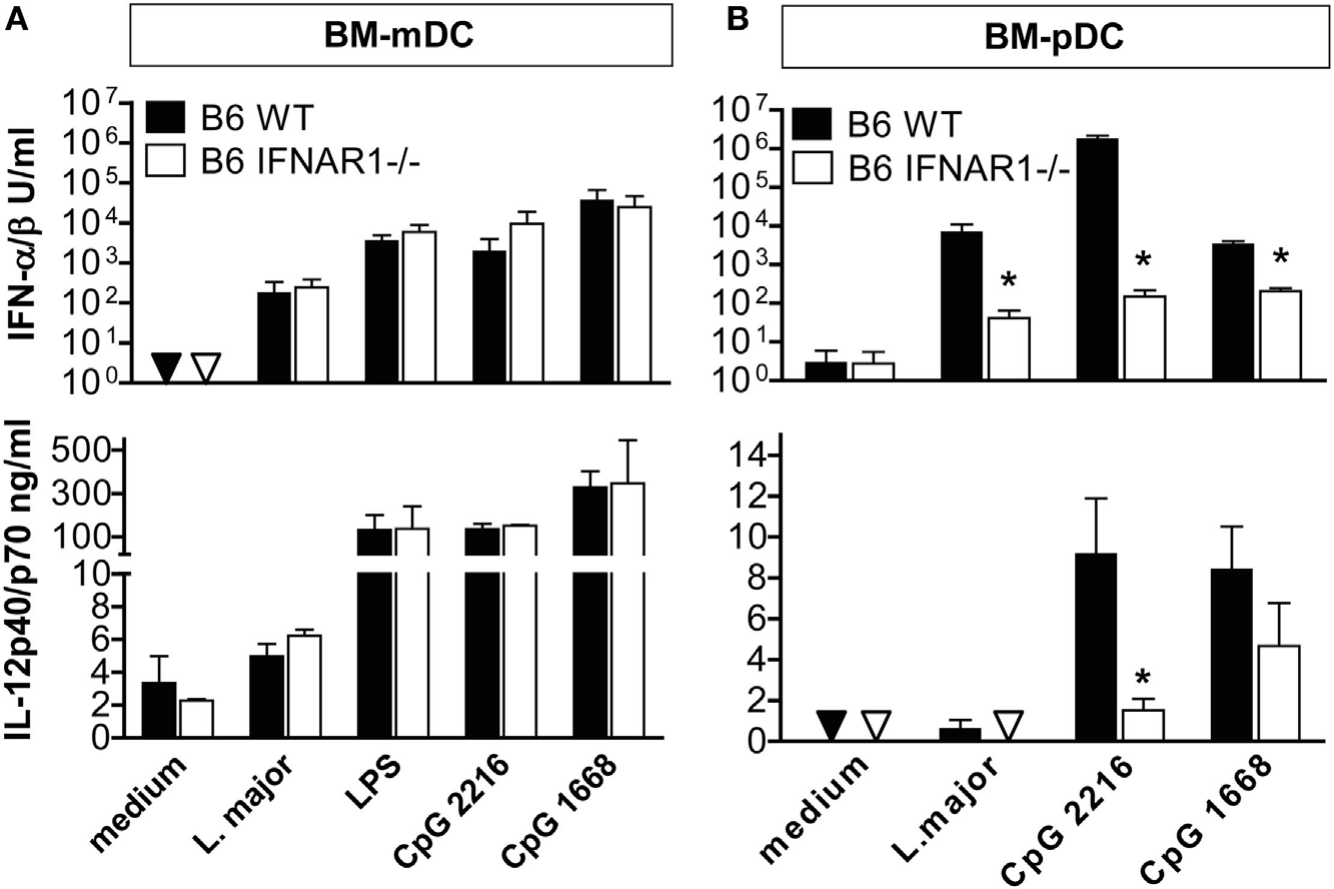
IFNAR signaling affects the *Leishmania major*-induced production of interferon (IFN)-α/β and interleukin (IL)-12 in BM-plasmacytoid dendritic cells (pDCs), but not bone marrow-derived dendritic cells (BM-mDCs). CD11b^+^CD11c^+^CD86^−^ sorted BM-mDCs and B220^+^CD11b^int^CD11c^+^ sorted BM-pDCs were cultured for 48 h with *L. major* promastigotes (multiplicity of infection = 3), 200 ng/ml LPS, 1 µM CpG-oligodesoxynucleotides (ODN) 2216 or CpG-ODN 1668. Culture supernatants were analyzed for IFN-α/β (vesicular stomatitis virus bioassay with L929 fibroblasts) and IL-12p40/p70 content (ELISA). Triangles depict values below the detection limit of the assays. **(A)** BM-mDCs, **(B)** BM-pDCs. Mean results (±SEM) of three to four independent experiments are shown. Asterisks depict significant differences between wild-type (WT) and IFNAR1^−/−^ cells (*p < 0.05; Mann–Whitney test).

The unaltered expression of IL-12p35 and IL-12p40 seen in *L. major*-infected C57BL/6 IFNAR1^−/−^ mice as compared to C57BL/6 WT mice (Figure 3) contrasts with a previous *in vitro* study in which type I IFN activity was required for optimal IL-12 expression by mouse bone marrow-derived dendritic cells (BM-mDCs) after stimulation with toll-like receptor (TLR) 3 plus TLR7 or TLR4 plus TLR7 agonists (48). We therefore tested whether the production of IL-12p40/p70 and IFN-α/β by BM-mDC in response to *L. major* promastigotes, a process that is triggered by TLR9 (15, 49), is dependent on endogenous IFNAR signaling. The TLR9 agonists CpG-B ODN 1668 and CpG-A ODN 2216 were used as control stimuli and mouse BM-pDC as control cells. In experimental leishmaniasis, mDCs are the key source of IL-12 (50–52) but weak producers of IFN-α/β (15), whereas pDCs generate considerably less IL-12, but copious amounts of IFN-α/β in response to *Leishmania* parasites (15). Both the IL-12 and the IFN-α/β production by BM-mDCs remained unaltered in the absence of IFNAR, irrespective of the stimulus used (Figure 5A). By contrast, in pDCs IFNAR signaling not only positively regulated the *Leishmania*- or CpG-induced production of IFN-α/β confirming our previous data (15), but was also necessary for maximal IL-12 release (Figure 5B).

From these data, we conclude that the *L. major*-induced production of IL-12 by mDCs, but not by pDCs, is fully preserved in the absence of type I IFN signaling.

### IFN-β or IFNAR1 Deficiency Does Not Alter the Course of Infection in Non-Healing BALB/c Mice

**Figure 6:**
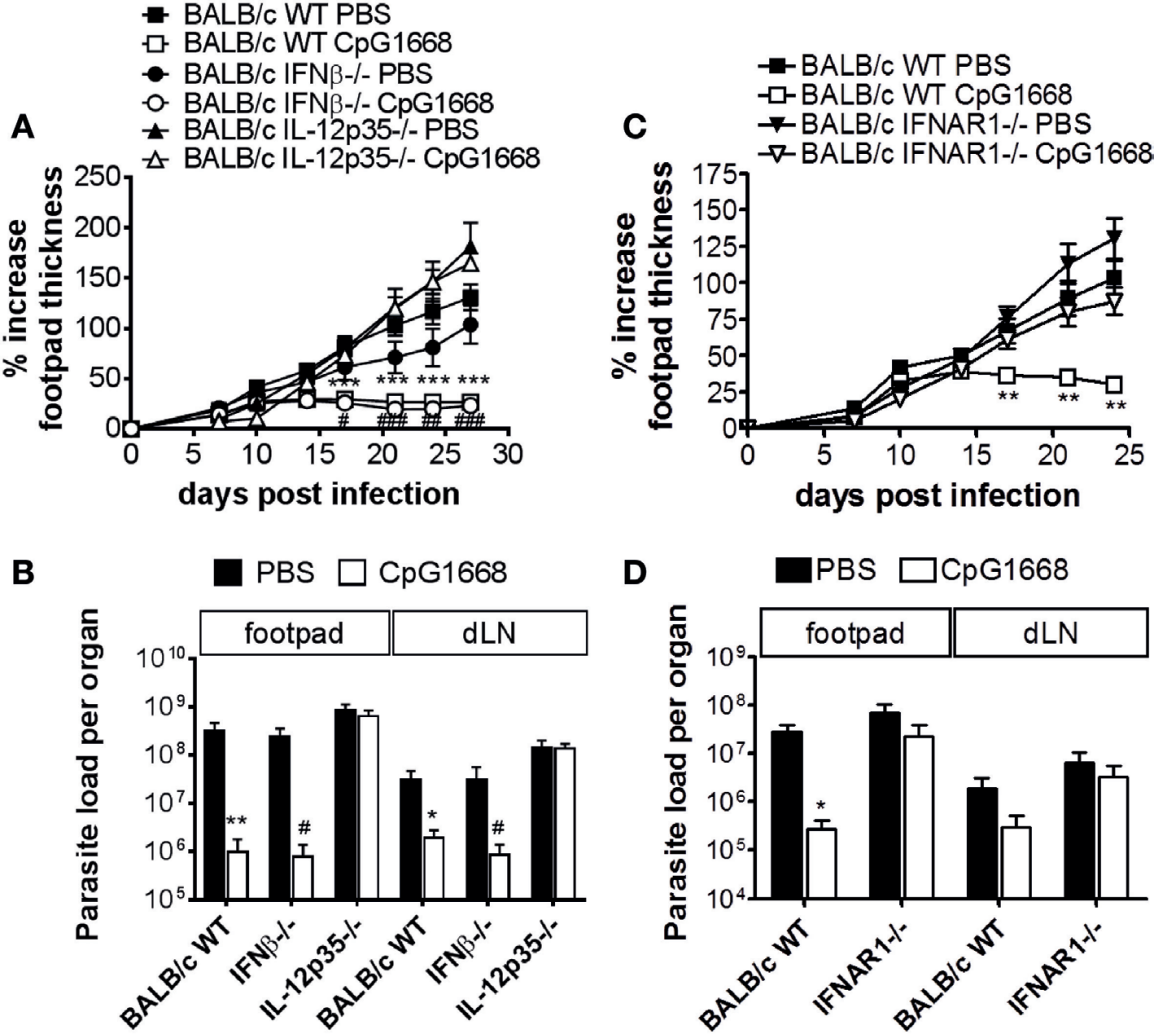
Course of *Leishmania major* infection in untreated or CpG-oligodesoxynucleotides (ODN) 1668-treated BALB/c wild-type (WT), interferon (IFN)-β^−/−^, IFNAR1^−/−^, and interleukin (IL)-12p35^−/−^ mice. Mice were infected with 3 × 10^6^ stationary phase *L. major* promastigotes into both hind footpads. 2 h before infection, 5 nmol CpG-ODN 1668 was administered subcutaneously at the site of infection, and 10 h postinfection additional 5 nmol CpG-ODN 1668 was injected intraperitoneally. **(A,C)** Development of footpad lesions in untreated vs. CpG-ODN 1668-treated BALB/c WT vs. IFN-β^−/−^ vs. IL-12p35^−/−^ or BALB/c WT vs. IFNAR1^−/−^ mice. The relative footpad thickness increase (mean ± SEM) of two to three independent experiments with three to five mice per group is shown. **(B,D)** Parasite burden in the footpads and draining lymph nodes of untreated vs. CpG-ODN 1668-treated BALB/c WT vs. IFN-β^−/−^ vs. IL-12p35^−/−^ or BALB/c WT vs. IFNAR1^−/−^ mice at day 27 (B) or day 24 (D) after infection. Mean results (±SEM) of two to three independent experiments with three to five mice per group are shown. Significant differences by Mann–Whitney test between PBS- and CpG-treated BALB/c WT mice (*p < 0.05; **p < 0.01; and ***p < 0.001) or PBS- and CpG-treated IFN-β^−/−^ mice (^#^*p* < 0.05; ^##^*p* < 0.01; and ^###^*p* < 0.001) are indicated.

**Figure 7:**
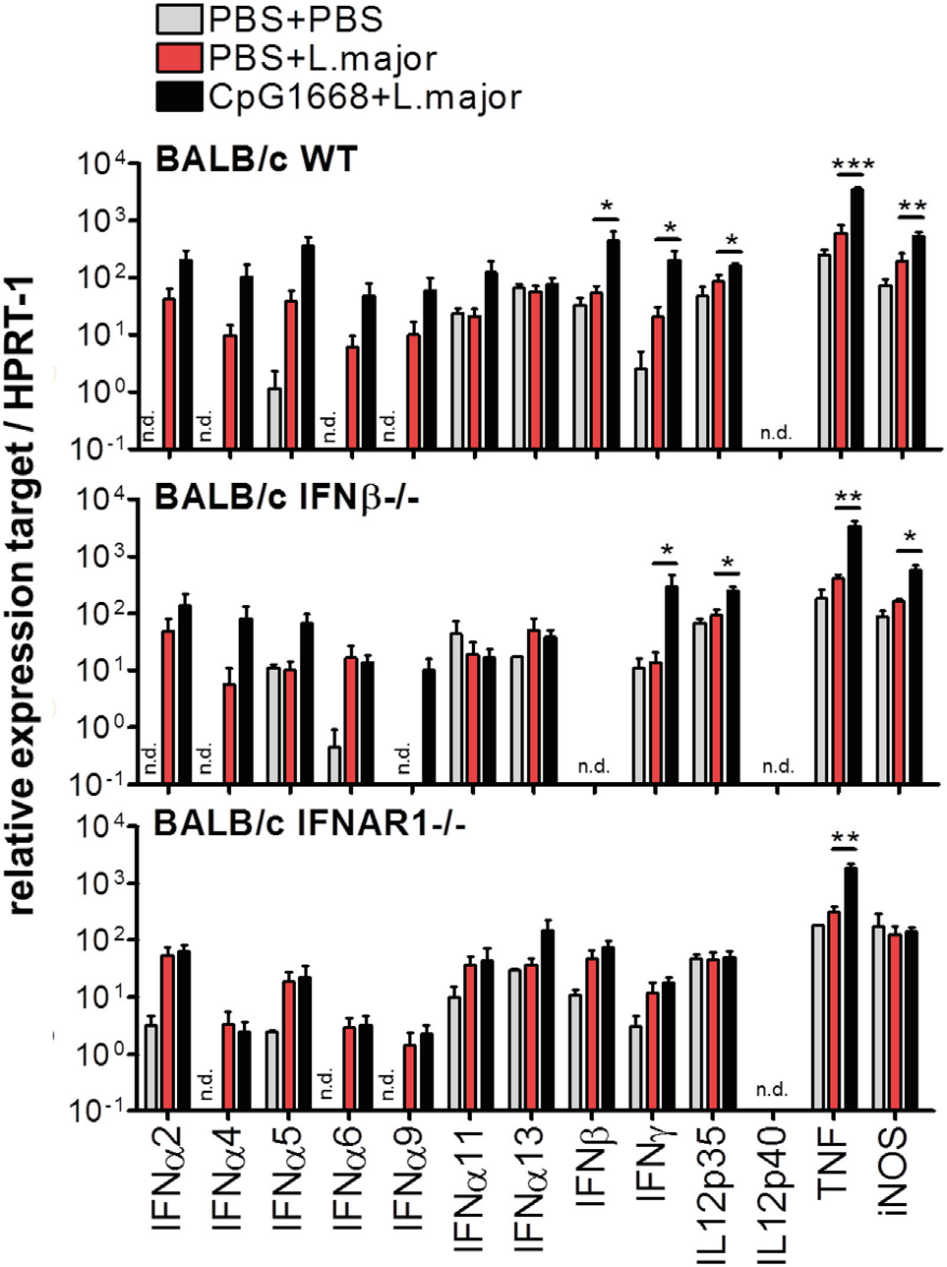
Effect of CpG-oligodesoxynucleotides (ODN) 1668 treatment on the cytokine mRNA expression in the footpad of *Leishmania major*-infected mice. BALB/c wild-type (WT), interferon (IFN)-β^−/−^ and IFNAR1^−/−^ mice were infected with 3 × 10^6^ stationary phase *L. major* promastigotes into both hind footpads or injected with PBS as control. 2 h before infection, 5 nmol CpG-ODN 1668 was administered subcutaneously at the site of infection, and 10 h postinfection additional 5 nmol CpG-ODN 1668 was injected intraperitoneally. Control mice received PBS. Total RNA was isolated from footpad tissue 36 h p.i. and reverse transcribed. Gene expression levels were determined as described in legend of Figure 3. Results are mean expression levels (±SEM) from three independent experiments with two to three mice per group. Significant differences by Mann–Whitney test between PBS- and CpG-treated infected BALB/c WT, IFN-β^−/−^, or IFNAR1^−/−^ mice (*p < 0.05; **p < 0.01; and **p < 0.001) are indicated. n.d., Not detectable.

In the light of the multiple suppressive functions of type I IFNs on DCs, macrophages, NK cells, and T cells [reviewed in Ref. (5–8)] and previous reports on disease-aggravating effects of type I IFNs in mouse and human infections with South American *Leishmania* species (24–26), we considered the possibility that type I IFNs might contribute to the non-healing pathology of cutaneous *L. major* infection observed in BALB/c mice. Infection of BALB/c IFN-β^−/−^ or IFNAR1^−/−^ mice, however, revealed that the clinical development of the skin lesions and the tissue parasite burden were not significantly different from WT BALB/c mice (Figures 6A,D), despite the rapid induction of various type I IFNs (i.e., IFN-α2, IFN-α4, IFN-α5, IFN-α6, and IFN-α9) at the site of infection in BALB/c WT mice (see Figure 7, upper panel, PBS/*L. major* vs. PBS/PBS). Thus, type I IFNs are unlikely to account for the lack of parasite control in BALB/c mice.

### The CpG-ODN Induced Protection of BALB/c Mice Is Preserved in the Absence of IFN-β, but Lost in IFNAR1 Deficiency

Finally, we addressed the question, whether the previously described protection of BALB/c mice from progressive cutaneous leishmaniasis following a prophylactic treatment with the CpG-ODN 1668 (23) is also observed when the type I IFN system is functionally impaired or blocked. As expected (23), injection of CpG-ODN 1668 before and shortly after infection prevented ulcerative skin lesions and restored parasite control in otherwise non-healing BALB/c WT mice. This was also the case in BALB/c IFN-β^−/−^ mice (Figures 6A,B). Equivalent results were obtained with CpG-ODN 2216 (data not shown). In BALB/c IL-12p35^−/−^ mice (Figures 6A,B) and in BALB/c IFNAR1^−/−^ mice (Figures 6C,D), however, the protective effect of CpG-ODN 1668 was lost. From these data, we conclude that IFNAR1 signaling (notably by IFN-α) and IL-12 are required for the immunoprophylactic activity of CpG-ODNs, whereas IFN-β is dispensable for the protective effect of CpG-ODNs.

To further ascertain the cooperation of type I IFNs and IL-12, we investigated whether the intracutaneous and intraperitoneal application of CpG-ODN 1668 indeed leads to a simultaneous upregulation of type I IFNs, IL-12, and possibly other cytokines (IFN-γ and TNF) that are known for their macrophage activating and protective effects in murine cutaneous leishmaniasis (53–56). In the footpads of *L. major*-infected WT BALB/c mice from three independent experiments, CpG-ODN 1668 led to a significant increase of IFN-γ, IL-12p35, TNF, and iNOS mRNA expression at 36 h after infection (i.e., 26 h after the last CpG-ODN 1668 injection) (Figure 7, upper panel). As seen in a previous study (49), IL-12 p40 mRNA was undetectable in whole organ RNA preparations from skin lesions, because at this early time point of infection the expression of IL-12 is restricted to a small number of DCs. With the notable exception of IFN-α13, CpG-ODN 1668 also enhanced the expression of type I IFNs in *L. major*-infected BALB/c WT mice, although the level of significance was only reached for IFN-β (Figure 7, upper panel). In *L. major*-infected BALB/c IFN-β-deficient mice, the stimulatory effect of CpG-ODN-1668 on the expression of IFN-α subtypes (α2, α4, α5, and α9), cytokines (IFN-γ, IL-12p35, and TNF), and iNOS expression was mostly maintained, whereas in IFNAR1-deficient BALB/c mice the upregulation of IFN-α subtypes, IFN-β, IFN-γ, IL-12p35, and iNOS was absent (Figure 7, middle and lower panel).

**Figure 8:**
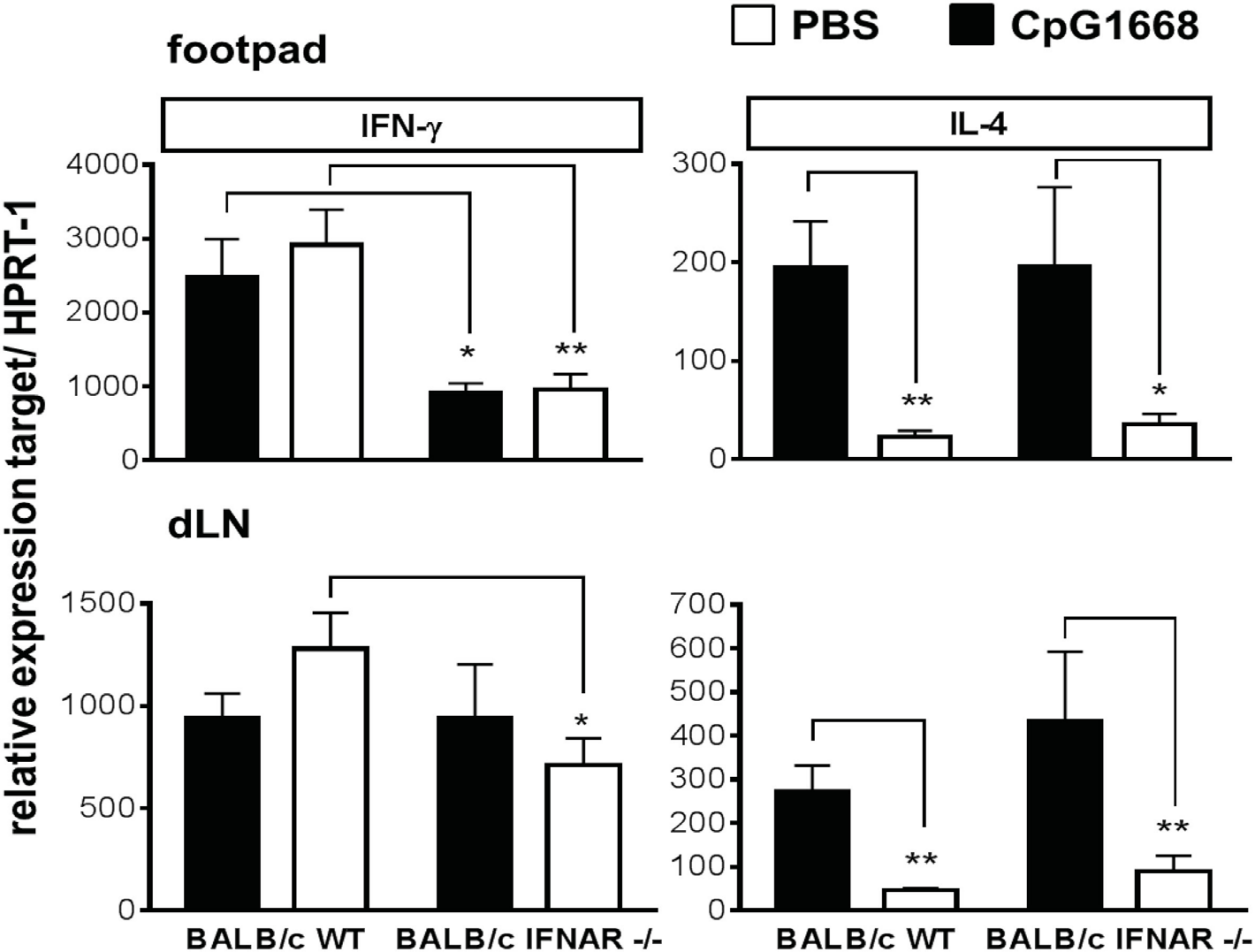
Interferon (IFN)-γ and interleukin (IL)-4 mRNA expression in the footpads and draining lymph nodes (dLN) of *Leishmania major*-infected BALB/c wild-type (WT) and IFNAR1^−/−^ mice treated with CpG-oligodesoxynucleotides (ODN) 1668. BALB/c WT and IFNAR1^−/−^ mice were infected with 3 × 10^6^ stationary phase *L. major* promastigotes into both hind footpads. 2 h before infection, 5 nmol CpG-ODN 1668 was administered subcutaneously at the site of infection, and 10 h postinfection additional 5 nmol CpG-ODN 1668 was injected intraperitoneally. Control mice received PBS (same volume). Total RNA was isolated from footpad and dLN tissue at day 23 or day 24 of infection and reverse transcribed. Gene expression levels of IFN-γ and IL-4 were determined as described in legend of Figure 3. Results are mean expression levels (±SEM) from two independent experiments with three mice per group. Significant differences by Mann–Whitney test (*p < 0.05 and **p < 0.01) are indicated.

Finally, we tested whether *L. major*-infected BALB/c WT mice and IFNAR1^−/−^ mice treated with PBS or CpG-ODN 1668 differed in their expression of IL-4 and IFN-γ mRNA at day 23 or day 24 of infection, when the lesions of CpG-ODN 1668-treated WT mice were strikingly smaller than of CpG-ODN 1668-treated IFNAR1^−/−^ mice (Figure 6C). As shown in Figure 8, treatment with CpG-ODN 1668 insignificantly upregulated the expression of IFN-γ mRNA in BALB/c WT mice. By contrast, in BALB/c IFNAR1^−/−^ mice, IFN-γ mRNA was strongly reduced as compared with WT mice and was not rescued by CpG-ODN 1668 treatment. In both strains of mice, CpG-ODN 1668 caused a strong suppression of IL-4 mRNA (Figure 8). Consequently, the IFN-γ/IL-4 mRNA ratio was approximately fourfold lower in CpG-ODN 1668-treated BALB/c IFNAR1^−/−^ mice as compared with CpG-ODN 1668-treated BALB/c WT mice.

Taken together, we conclude that CpG-ODN 1668 prevented progressive disease in BALB/c WT mice by (a) boosting the early expression of iNOS and several protective cytokines (IFN-α subtypes, IFN-γ, IL-12p35, and TNF) and (b) by downregulating IL-4, which—except for the effects on TNF and IL-4—required an intact IFNAR signaling. Therefore, CpG-ODN 1668 failed to convey protection in BALB/c IFNAR1^−/−^ mice.

## Discussion

During infections of mice with bacteria, protozoa, or fungi, endogenously produced type I IFNs either supported or impeded the control of the pathogens and the survival of the animals (57–78). In this study, we found that the self-healing course of infection seen in *L. major*-infected C57BL/6 WT animals was fully preserved in C57BL/6 mice lacking the IFN-β-gene or a functional type I IFN receptor (IFNAR1^−/−^). In genetically susceptible BALB/c mice, which develop progressive skin lesions after cutaneous infection with *L. major* and ultimately succumb to visceral disease, a deficiency of IFN-β or IFNAR also did not alter the clinical outcome of the infection. However, the protective effect of a treatment of otherwise non-healing BALB/c mice with CpG-containing ODNs was completely abolished in the absence of type I IFN signaling. These results raise important questions on the regulation and function of type I IFN expression during different infections and *in vivo* conditions.

### Type I IFN Expression during Non-Viral Infections

Very few studies have addressed the expression of type I IFNs in response to pathogens other than viruses *in vivo*. Several groups measured IFN-α/β bioactivity or IFN-β protein in the serum of mice infected with bacteria or protozoa, but quantitative type I IFN subtype analyses *in vivo* during the course of infection are lacking [reviewed in Ref. (6, 10, 11)]. In the C57BL/6 *L. major* infection model studied here, we found a rapid and striking induction of IFN-β mRNA in the infected skin, to a lesser degree also of IFN-α4 and IFN-α5 (Figure 1). Based on results obtained with viral infections of cell lines, IFN-β and IFN-α4 (and presumably also IFN-α5) act as immediate-early IFNs that are elicited by activation of the transcription factor interferon-regulatory factor (IRF)-3 and IRF-7 and account for the subsequent amplification of the type I IFN response *via* crosslinking of IFNAR (41). IFN-α13 has been originally described as an IFN-α subtype that is expressed in mouse fibroblast cell lines even in the absence of a viral stimulus or an IFN-α/β priming signal (79). In this study, we found that the constitutive expression of IFN-α13 (and of IFN-α11) in naïve mice is subject to negative regulation following *L. major* infection. Looking at the entire course of infection, IFN-α13 and IFN-β are reciprocally regulated and expressed, which suggests that both subtypes fulfill different functions. In the human type I IFN system, there is evidence for a functional diversity of IFN-α/β subtypes [reviewed in Ref. (80)]. The underlying structure–function relationships are only beginning to emerge (81, 82). In the mouse system, we are still largely lacking important tools (recombinant proteins, subtype-specific antibodies, and knockout mice) to investigate the function of the different IFN-α/β subtypes.

### Function of Endogenous Type I IFN during Non-Viral Infections

IFNAR1^−/−^ mice infected with group B streptococci, *Streptococcus pneumoniae, Escherichia coli* (65), *Trypanosoma cruzi* (61, 62), *Plasmodium yoelii* (74), *Plasmodium berghei* (83), *Pneumocystis carinii* (84), or *Cryptococcus neoformans* (64) all showed a significantly enhanced pathogen load and/or reduced survival compared with the respective WT control mice. The protective effect of type I IFN seen in these models is contrasted by studies on other infectious pathogens, in which type I IFN signaling was either clearly associated with reduced pathogen control, striking tissue damage and increased mouse mortality [e.g., *Listeria monocytogenes* (28, 58, 59, 69), *Mycobacterium tuberculosis* (60, 63, 71, 85), *Staphylococcus aureus* (67), and *Candida albicans* (72)] or without strong impact on the course of infection at all [e.g., *Legionella pneumophila* (86)]. These findings strongly suggest that the diverse immunomodulatory activities of type I IFNs are either beneficial or detrimental for the host, depending on the eliciting infectious agent. Even within one pathogen species (i.e., *T. cruzi, M. tuberculosis*, and *P. berghei*) opposing functional roles of type I IFNs (mediating resistance vs. susceptibility) have been described depending on the pathogen strain and infection dose (62, 70), the genetic mouse model used (71, 77), or the time points of infection analyzed (73, 83).

A similar complexity of the activity of type I IFNs is also seen in experimental cutaneous leishmaniasis, where the infection site, the parasite inoculum, and the parasite species and strain are known to affect the immune response and course of infection [reviewed in Ref. (17, 18)]. As shown in the present analysis of *L. major*-infected C57BL/6 mice, parasite and disease control was unaffected in the absence of IFNAR signaling. At first sight, this result was unexpected considering the striking impact of type I IFNs during the NK cell phase of *L. major* infection (days 1 and 2) (21) and the sustained expression of certain type I IFNs during the course of infection documented here for the first time (Figure 1). However, the non-essential role of type I IFNs in this model is most likely due to the fact that the swiftly starting production of IFN-γ by NK cells and recruited CD4^+^ T cells [reviewed in Ref. (87)] makes type I IFNs rapidly dispensable as inducers of iNOS, also because IFN-γ is considerably more potent than type I IFNs in triggering this antileishmanial effector mechanism (88). In addition, at least *in vitro* the production of high amounts of type I IFNs in response to *L. major* parasites appeared to be restricted to pDCs (15), which represent a minor cell population *in vivo*, whereas the more abundant mDCs and macrophages released considerably smaller or very low amounts of IFN-α/β, respectively (21, 89) (Figure 5A vs. Figure 5B). Furthermore, the production of IL-12 by mouse mDCs, which is crucial for eliciting a Th1 response, was completely independent of IFNAR1 signaling (Figure 5A, lower panel), in contrast to recent findings with human DCs (90). Finally, *L. major* parasites impeded the expression of various type I IFN subtypes in macrophages (89) and were reported to produce a mammalian casein kinase 1-ortholog, which modestly decreased the expression of IFNAR1 on the surface of mouse BM macrophages and human DCs (91). All these factors might limit the expression and/or function of type I IFNs in *L. major*-infected C57BL/6 mice.

In *L. amazonensis*-infected 129Sv mice, which develop non-healing, progressive skin lesions, deletion of IFNAR1 was associated with a markedly attenuated clinical course of infection and parasite load. Absence of IFNAR1 led to an increased recruitment and death of neutrophils, which upon interaction with macrophages facilitated the killing of parasites (25). By contrast, in *L. mexicana*-infected mice, another model for non-healing cutaneous leishmaniasis, the lesion development and parasite burden were comparable in WT and IFNAR^−/−^ mice on a mixed 129Sv/C57BL/6 background, with only a transient defect of the production of IFN-γ and IL-10 upon *in vitro* restimulation of lymph node T cells (92). Strains of *Leishmania (Viannia) guyanensis*, which carried high amounts of *Leishmania* RNA virus-1 (LRV1) and elicited metastatic skin lesions, activated macrophages for the release of much higher levels of IFN-β compared with non-metastatic parasite strains that lacked the virus (93). IFNAR1^−/−^ mice infected with LRV1-positive strains of *L. V. guyanensis* developed markedly attenuated skin lesions compared with WT control mice, demonstrating that in chronic non-healing cutaneous leishmaniasis exuberant amounts of type I IFNs (triggered by the activity of the *Leishmania* RNA virus) are counterprotective (26). In line with these observations, exogenous IFN-β impeded the killing of *Leishmania (Viannia) braziliensis* or *L. amazonensis* by human macrophages *via* induction of superoxide dismutase 1 and subsequent degradation of ${\text{O}}_{2}^{-}$ (24, 94). By contrast, the quantities of type I IFNs generated in BALB/c mice infected with *L. major* were not sufficient to impair the immune response as revealed by the unaltered course of infection in BALB/c IFNAR1^−/−^ mice.

Finally, in mouse visceral leishmaniasis caused by *Leishmania donovani*, the parasite burden in liver and spleen was reported to be unaffected in the absence of IFNAR signaling, but original data were not presented in this publication (95). The reduced parasite control observed in *L. donovani*-infected mice deficient for either the interferon-regulatory factor-5 (IRF-5) or IRF-7, both of which control type I IFN expression, was attributed to an impaired generation of Th1 cells and IFN-γ and a defective induction of iNOS (96, 97).

### Mechanism of CpG-Induced Protection

The finding that CpG-ODN 1668 conferred protection equally well in *L. major*-infected BALB/c WT and IFN-β^−/−^ mice, but was ineffective in both BALB/c IFNAR1^−/−^ and BALB/c IL-12p35^−/−^ mice, suggested that IFN-β is either irrelevant or that its function can be fully compensated by IFN-α subtypes and/or that IFNAR signaling might affect the CpG-induced expression of protective cytokines other than IFN-α subtypes. Indeed, in IFN-β^−/−^ mice, the CpG-induced upregulation of certain IFN-α subtypes (α2, α4, α5, and α9) and of IFN-γ mRNA was completely preserved (Figure 7, middle panel). By contrast, in IFNAR1^−/−^ mice CpG-ODN 1668 failed to boost the mRNA expression of most IFN-α subtypes as well as of IFN-β, IFN-γ, and IL-12p35 (Figure 7, lower panel), whereas the CpG-ODN 1668-mediated suppression of IL-4 was maintained in the absence of IFNAR1 (Figure 8). IFNAR signaling is known to amplify the expression of type I IFNs (especially IFN-α) in a positive feedback loop (12–15). Thus, CpG-ODN 1668 is likely to protect BALB/c mice from non-healing disease *via* the induction of IFN-α, IFN-γ, and IL-12 and the ensuing expression of iNOS.

In summary, this study shows that several members of the type I IFN family are prominently expressed and regulated during *L. major* infection. Although type I IFNs are dispensable for the spontaneous cure of primary or secondary *L. major* infections in self-healing mice, their disease-preventive activity can be readily revealed during CpG-ODN-induced protection of otherwise non-healing BALB/c mice. However, previous reports on other non-viral infections illustrated that the function of type I IFNs clearly varies with the pathogen (also within the genus *Leishmania*) and ranges from resistance-mediating to pathology- and disease-promoting. This needs to be considered when type I IFNs are applied for the treatment of chronic viral infections, autoimmune diseases, or malignancies in patients with persistent bacterial or parasitic infections.

## Ethics Statement

Animal housing and experimental studies were approved by the local or governmental authorities in Freiburg and Ansbach.

## Author Contributions

US, JL, NJ, SH, and HS designed, performed, and analyzed experiments, interpreted data, and prepared the figures. TM and HS performed and analyzed experiments. UK and SW provided mice and helped to interpret data. CB designed experiments, interpreted the data, and wrote the manuscript. All the authors approved the final version of the manuscript.

## Conflict of Interest Statement

The authors declare that the research was conducted in the absence of any commercial or financial relationships that could be construed as a potential conflict of interest.

## Abbreviations

BM, bone marrow; BM-DC, bone marrow-derived dendritic cell; (c, m) DC, (conventional or myeloid) dendritic cell; iNOS (NOS2), inducible (or type 2) nitric oxide synthase; NK, natural killer; pDC, plasmacytoid dendritic cell; SN, supernatant.
