# Supplementary material for: Type I Interferon Signaling Is Required for CpG-Oligodesoxynucleotide-Induced Control of Leishmania major, but Not for Spontaneous Cure of Subcutaneous Primary or Secondary L. major Infection
Source: Front Immunol. 2018 Feb 5;9:79. doi: 10.3389/fimmu.2018.00079 (PMC5807663; doi:10.3389/fimmu.2018.00079)
Supplement: Supplementary file 1 [file Image_1.PDF]

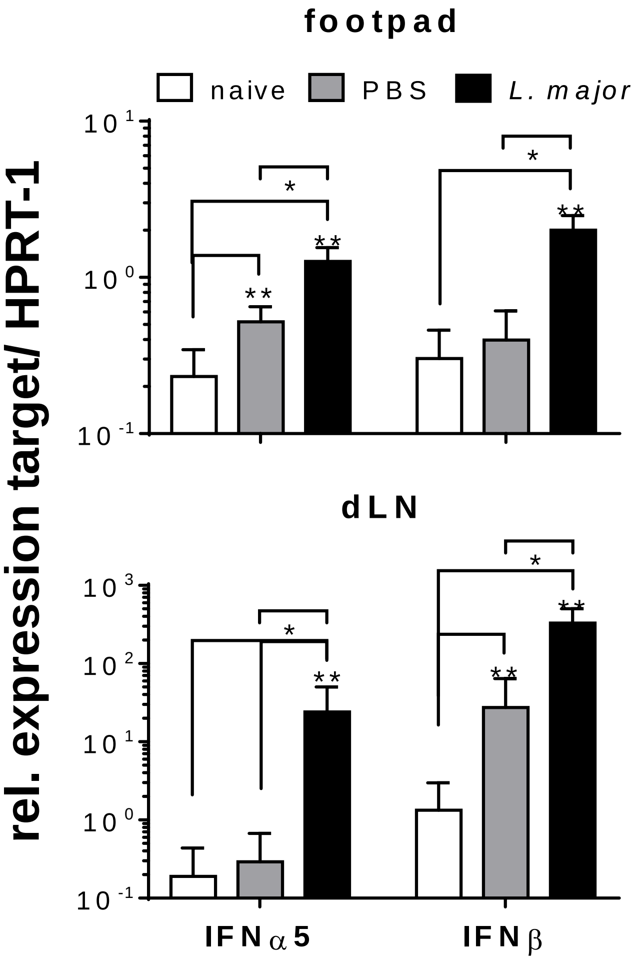

Supplemental Fig. 1 Upregulation of IFNα5 and IFNβ in *L. major*-infected C57BL/6 mice at day 2 of infection. mRNA expression of IFN-α5 and IFN-β in footpad tissue or the draining lymph nodes of naïve C57BL/6 mice, C57BL/6 mice infected with 3×10^6 stationary phase *L. major* promastigotes (in 50 µl PBS) or control mice injected with 50 µl PBS alone was analyzed at day 2 p.i. using quantitative RT-PCR. Results are shown as mRNA levels (mean ± SEM) normalized to the endogenous control HPRT-1 from 2 independent experiments (2-3 mice per group and time point, respectively). Asterisks represent the respective significance values (*, $p<0.05$; **, $p<0.01$; Mann-Whitney test).
